# Implications of the Dependence of Neuronal Activity on Neural Network States for the Design of Brain-Machine Interfaces

**DOI:** 10.3389/fnins.2016.00165

**Published:** 2016-04-20

**Authors:** Stefano Panzeri, Houman Safaai, Vito De Feo, Alessandro Vato

**Affiliations:** Neural Computation Laboratory, Istituto Italiano di TecnologiaRovereto, Italy

**Keywords:** brain-machine interfaces, neuromodulation, neural coding, state dependence, neural response variability

## Abstract

Brain-machine interfaces (BMIs) can improve the quality of life of patients with sensory and motor disabilities by both decoding motor intentions expressed by neural activity, and by encoding artificially sensed information into patterns of neural activity elicited by causal interventions on the neural tissue. Yet, current BMIs can exchange relatively small amounts of information with the brain. This problem has proved difficult to overcome by simply increasing the number of recording or stimulating electrodes, because trial-to-trial variability of neural activity partly arises from intrinsic factors (collectively known as the network state) that include ongoing spontaneous activity and neuromodulation, and so is shared among neurons. Here we review recent progress in characterizing the state dependence of neural responses, and in particular of how neural responses depend on endogenous slow fluctuations of network excitability. We then elaborate on how this knowledge may be used to increase the amount of information that BMIs exchange with brain. Knowledge of network state can be used to fine-tune the stimulation pattern that should reliably elicit a target neural response used to encode information in the brain, and to discount part of the trial-by-trial variability of neural responses, so that they can be decoded more accurately.

## Introduction

Brain-machine interfaces (BMIs) are devices mediating the dialogue between a brain and the external world. These devices hold the potential to restore motor or sensory functions to people who lost them due to illness or injury. Depending on their direction of communication with the brain, BMIs can be divided into various categories (Donoghue, 2002; Mussa-Ivaldi and Miller, 2003).

*Efferent or motor BMIs* use sensors to record neural activity—such as single-unit (SUA) or Multi-unit (MUA) activity, Local Field Potentials (LFPs), electrocorticograms (ECoG), or electroencephalograms (EEGs)—and decode this activity to infer the motor intent of the subject and command an artificial actuator (a robotic arm, a motorized wheelchair, or a computer cursor). These systems can have a considerable clinical impact for the treatment of patients with neurological diseases such as stroke, spinal cord injury, or Parkinson's disease.

*Afferent or sensory BMIs* sense physical quantities from the environment (i.e., sound, light, temperature) and use an encoding interface to translate these sensory signals into patterns of neural activity elicited using causal interventions on the brain (for example, electrical or optogenetic microstimulation) with the goal of provoking the desired sensation (Fitzsimmons et al., 2007). Examples include cochlear implants (Loeb, 1990; Clark, 2006) and retinal prostheses (Zrenner, 2002; Nirenberg and Pandarinath, 2012). Sensory interfaces have obvious implications for curing the loss of sensory function.

**Figure 1:**
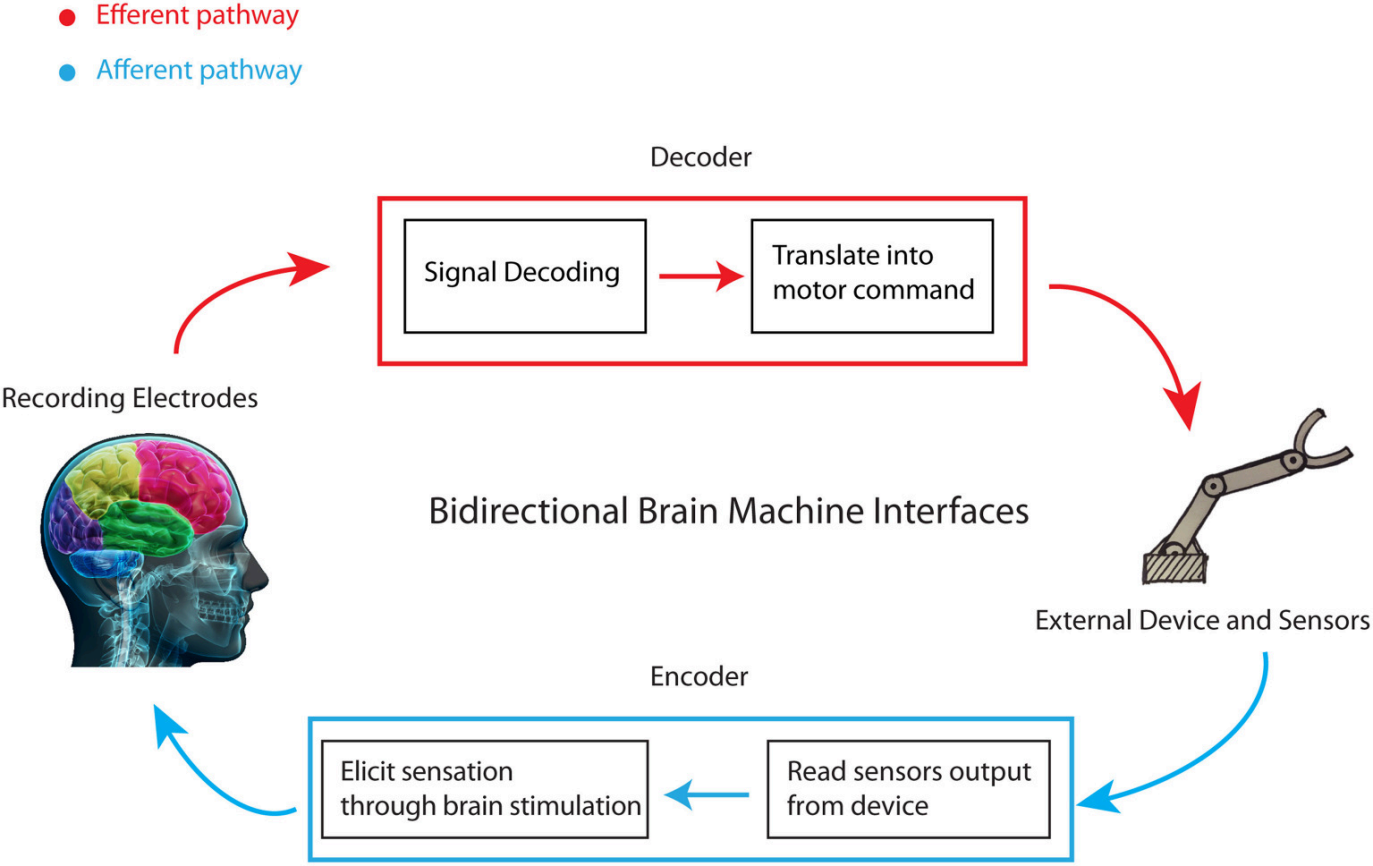
**Schematic of a bidirectional brain-machine interface**. A bidirectional BMI has two pathways of communication with the brain: an afferent pathway from some sensors to the brain and an efferent pathway from the brain to a device controlled by it. The decoder—or motor interface—transforms the recorded activity into motor commands for the device. The encoder—or sensory interface—transmits the information about the external world or about the state of the device to the brain by delivering electrical stimulation patterns to it.

Researchers have also developed *bidirectional BMIs* (Figure 1) in which both a decoder of motor intention and an encoder of sensory information exchange information with the brain in a closed-loop (Reger et al., 2000; Nicolelis, 2003; Lebedev and Nicolelis, 2006; Nicolelis and Lebedev, 2009; O'Doherty et al., 2009, 2011; Mussa-Ivaldi et al., 2010; Lebedev et al., 2011; Carmena, 2013; Moxon and Foffani, 2015). Such systems may have important clinical applications because (unlike motor BMIs) they can provide the brain with non-visual feedback information (such as tactile or proprioceptive information) that is important for compliant task execution. Bidirectional BMIs may also help to automatically execute tasks without focusing attention on each single motor command. Recently we proposed to achieve this goal through a class of bidirectional BMIs, implemented both in anesthetized and awake rodents (Vato et al., 2012, 2014; Boi et al., 2015), in which the decoding and encoding interfaces generated a motor program similar to the force fields generated by the spinal cord when combining motor and sensory information (Shadmehr et al., 1993; Mussa-Ivaldi et al., 1994).

Despite all this progress, several aspects of current unidirectional and bidirectional BMIs remain to be improved (Bensmaia and Miller, 2014; Shenoy and Carmena, 2014). One key challenge is that the large trial-to-trial variability of neural responses (Faisal et al., 2008; Quiroga and Panzeri, 2009) strongly limits BMIs. Improving sensory interfaces requires converting more reliably a sensory signal into desired patterns of neural activity that can be robustly perceived as the appropriate sensory signal. Improving motor interfaces requires better decoding the motor intention despite the trial-to-trial variability of the neurons that express it. Variability of neural activity cannot be easily reduced simply by improving technology to record and stimulate from ever increasing numbers of electrodes (Baranauskas, 2014; Lebedev, 2014), because some of the main sources of variability are generated at the network level and are shared across neurons (Goris et al., 2014; Lin et al., 2015; Schölvinck et al., 2015). This can be conceptualized by thinking of neural activity as *state-dependent*: neural activity does not depend only on external task-related variables but also on internal network variables. In this Perspective article, we will discuss recent findings about state dependence of neural activity, and we will reason on how taking state dependence of neural activity into account can help us to build better sensory, motor, and bidirectional BMIs.

## State dependence of neural responses

Neural responses to a sensory stimulus do not only depend on the feedforward extrinsic sensory inputs but also on intrinsic network variables that can be collectively defined as the “neural state” (Buonomano and Maass, 2009). This state dependence is generated by strong recurrent and feedback connectivity that creates endogenous ongoing activity and modulates how afferent information is processed (Harris and Thiele, 2011). Similarly, firing of neurons in motor areas reflects not only the tuning to the movement being expressed but also other factors including ongoing network dynamics (Rule et al., 2015). In addition, neuromodulatory inputs from brainstem nuclei can modulate the dynamics of cortical networks (Moxon et al., 2007; Edeline, 2012; Eschenko et al., 2012; Lee and Dan, 2012).

That neural firing is state-dependent has been known for many years (Arieli et al., 1996). Recent years, however, have witnessed important progress in the mathematical understanding of how the single-trial neural responses to a particular stimulus or external event depend on network state. Biophysical models can predict the contribution to single-trial responses of individual neurons to electrical or optical stimulations arising from intrinsic neural mechanisms (such as adaptation) that can be inferred from the previous spiking history of that neuron (Ahmadian et al., 2011). These methods can in principle be extended to predict in real-time the optimal stimulation intensity needed to elicit a target pattern of neural activity while minimizing stimulation power (Ahmadian et al., 2011). Other theoretical work concentrated on mathematically predicting single-trial cortical activity elicited by a sensory stimulus by using a dynamical system to model the interaction between the feed-forward stimulus drive and the ongoing fluctuations of the circuit's state (Curto et al., 2009). This prediction worked well both when the interaction between ongoing state dynamics and stimulus drive is linear and when it is non-linear. Further work has shown that the prediction of single-trial cortical responses to stimuli can be greatly enhanced when knowledge of the state of neuromodulatory brainstem nuclei (in particular of the nucleus releasing norepinephrine) is used to improve the prediction of the ongoing cortical state dynamics (Safaai et al., 2015). A number of other recent studies clarified that the variability induced by state changes can be, to a first approximation, described simply. In some cases state dependence is described with an additive term of background activity to the trial-averaged response to the stimulus (Arieli et al., 1996; Ecker et al., 2014; Schölvinck et al., 2015). In other cases, state dependence can be described as multiplicative (i.e., it rescales the gain of the stimulus-response function, see Goris et al., 2014) or as a mixture of additive and multiplicative effects (Kayser et al., 2015; Lin et al., 2015). Importantly, the trial-to-trial variations of neural responses due to state changes are shared across neurons (Lin et al., 2015), likely because they arise as “network effects” (Harris and Thiele, 2011). Because it is shared, the variability due to state changes cannot be easily eliminated simply recording from more neurons.

Recent studies have begun individuating which variables describing intrinsic brain activity can be used as effective “neural state” variables. Several studies have suggested that cortex undergoes periodic endogenous slow periodic variations in excitability that can be captured by the phase of low-frequency activity of mass signals such as LFPs or MUA. For example, both in anesthetized (Kayser et al., 2015) and awake animals (Lakatos et al., 2005) certain phases of low-frequency LFP oscillations correspond to higher firing rate and other phases correspond to lower firing rate of single neurons. A recent study (Kayser et al., 2015) in anesthetized animals showed that the phase of low-frequency (delta, theta, and alpha) LFP oscillations at which a stimulus is presented rescales both the stimulus-response gain and the background firing of auditory cortical neurons. Similarly, the gain of visual cortical neurons of awake attentive macaques is modulated by intrinsic state variables varying on time scales of few hundreds ms (Rabinowitz et al., 2015). These low-frequency oscillations also correlate with perception: the phase of low-frequency (delta, theta bands) EEGs at which a near-threshold sensory stimulus is presented to human subjects impacts on whether the subject reports the perception of the stimulus (Busch et al., 2009; Ng et al., 2012). Moreover, the perception elicited by non-invasive transcranial stimulation of sensory cortices depends on the endogenous alpha EEG rhythm at the time of stimulation (Romei et al., 2008) suggesting that both the neural activity and the perceptual effect elicited by causal intervention in the awake brain depends on the ongoing endogenous low-frequency activity.

## How can state dependence of neural responses be used to improve BMIs?

Here we discuss how taking state dependence into account could increase the information bandwidth by which BMIs communicate with the brain.

**Figure 2:**
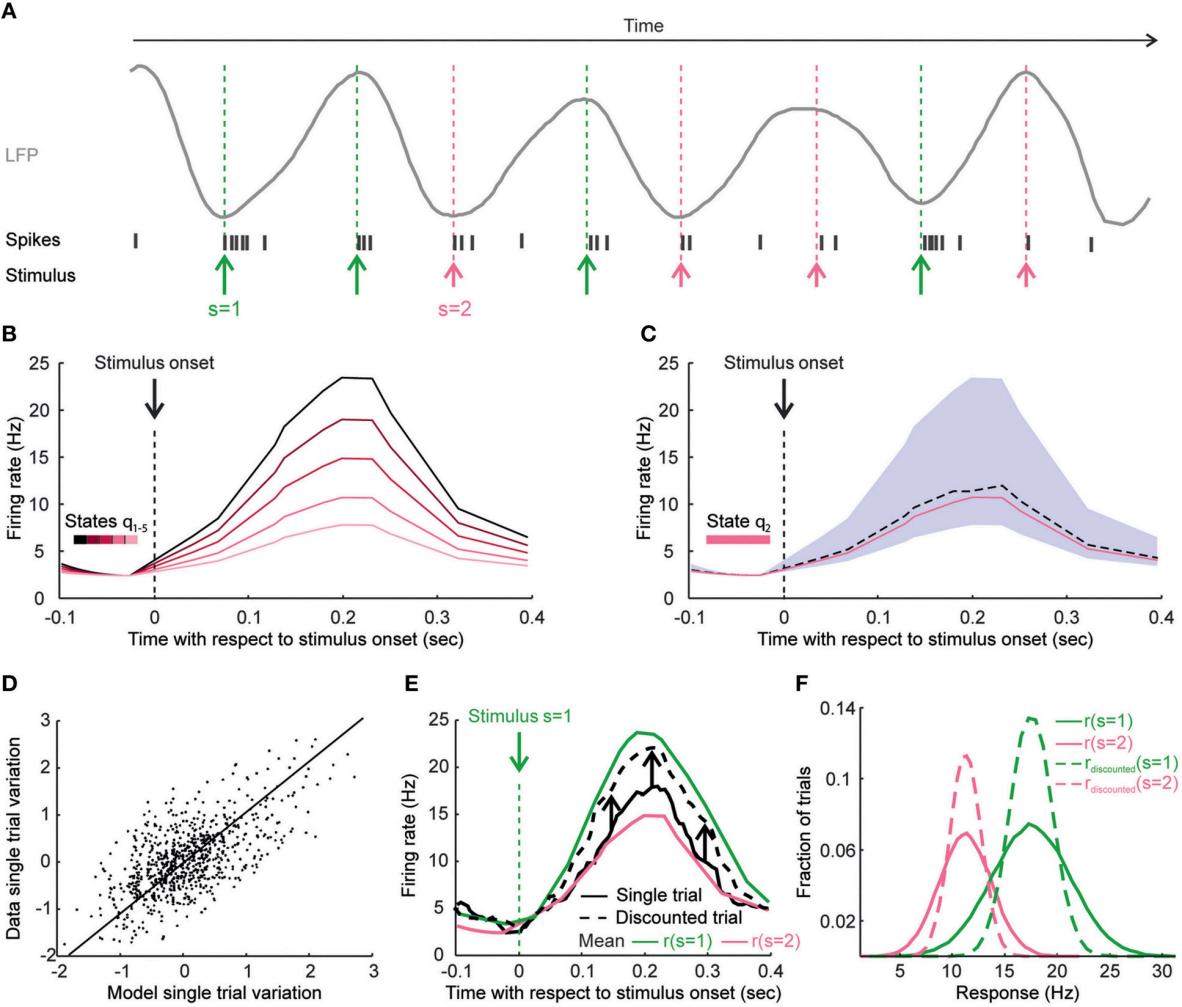
**How knowledge of state dependence may be used to improve reliability of elicited patterns and to enhance decoding of neural activity**. This figure uses cartoons of neural responses to illustrate how taking into account state dependence of neural responses may improve BMIs. **(A)** The panel illustrates the responses of a cartoon neuron to two different stimuli presented at different phases of LFP. Green and pink arrows represent the application times of stimulus *s* = 1 and *s* = 2, respectively. Stimuli applied at the trough of the LFP (a more excitable network state phase) elicit a stronger response than stimuli applied at the peak of the LFP (a less excitable network state phase). **(B)** We plot the time course of cartoon neural activity shortly after a stimulus is applied at time *t* = 0. Different lines represent single-trial responses to the same stimulus that were elicited in trials that differed by the value of the state variable (in this case, the LFP phase) in each trial. Each line representing the single-trial responses is color-coded by the value of the state variable in that trial. The response to the same stimulus has large trial-to-trial variability because of the difference in the state at which the stimulus is presented. **(C)** In a particular network state, the real response (dashed black line) and the state-dependent model prediction (pink line) of the response are shown. If the model of state dependence is accurate, it will help the experimenter to predict which response will be elicited in that trial given the stimulation parameters and the neural state, thereby narrowing down the uncertainty about which response will be elicited. The gray area shows the range of possible responses that could be obtained for a given stimulus because of state variations. **(D)** A scatter plot showing the variations around the mean of single-trial responses plotted against the single-trial response variations around the mean estimated from a state-dependent model of the responses in a hypothetical case. Each point represents one trial. This scatter plot indicates how well a state dependence model can predict the single-trial neural responses to a given stimulus. **(E)** How to discount state-induced trial-to-trial variability is exemplified for a single trial. Stimulus 1 was presented in this cartoon trial, and the response in this trial (full black line) is plotted against the trial-averaged response of stimulus 1 (green line) and of stimulus 2 (pink line). The state dependence model predicts that the response variation around the trial-average in this trial was negative. The black arrows show the model-predicted state-induced variation around the trial-average for this particular single trial. The addition of the model predicted variability gives a “discounted” response (dashed black line) that will be much closer to the averaged response to stimulus 1 (the stimulus actually presented in this trial) that the original response. **(F)** The distributions of the responses to stimulus 1 (green full line) and stimulus 2 (pink full line) and the distributions of the discounted responses for stimulus 1 (dashed green line) and stimulus 2 (dashed pink line) obtained after subtracting the model predicted state-induced variability are shown. The distributions of the discounted responses are narrower and allow better discrimination between the two stimuli.

To discuss this, we will suppose for simplicity that, as in auditory cortex (Kayser et al., 2015), the response *r* of a neuron to a stimulus *s* in a single trial *tr* depends on a state variable θ (in this example, the phase of a low-frequency LFP at which stimulus *s* is applied in trial *tr*) with an additive-multiplicative model. The gain *g* and the background *b* both depend on the phase of a low-frequency network oscillation at which the stimulus is applied

(1) $$\begin{array}{l} r\left(s,tr\right)=g\left(\theta \right)f\left(s\right)+b\left(\theta \right) \end{array}$$

where state θ is function of the trial and *f(s)* is the trial-averaged responses to all trials to stimulus *s* [in other words, *f(s)* is the neuron tuning's curve]. We assume that, again as in Kayser et al. (2015), if the stimulus was presented in the phases of the low-frequency LFP that corresponded to LFP troughs (respectively, peaks) then it elicited a larger (respectively, smaller) response because both the gain and the background activity were larger (respectively, smaller), see Figure 2A.

Knowledge of state dependence can improve the encoding stage of BMIs. If (as illustrated in Figure 2B) trial-to-trial variations of stimulus-evoked responses can be to some extent predicted from models of state dependence such as those in Equation (1), these model predictions can lead to design a “state-dependent” causal intervention onto the neural tissue to achieve more reliably the desired neural response (Figure 2C). Suppose that we want to achieve a target firing rate r^*^ on a given trial and that we estimate the network state at that time to be θ. Using Equation (1), the optimal value of the stimulation strength f^*^(*s*) that we need to apply to achieve the target response is:

(2) $$\begin{array}{l} f^{*}\left(s\right)=\frac{r^{*}-b\left(\theta \right)}{g\left(\theta \right)} \end{array}$$

The potential advantage of using knowledge of the state at which the stimulus is applied to better predict the responses that will be elicited has been tested *in vivo*. Brugger et al. (2011) successfully used the low-frequency (< 20 Hz) components of pre-stimulus LFPs to better predict the intensity of electrical microstimulation needed to achieve a target value of cortical firing in response to the stimulation. The algorithm was particularly successful at increasing the reliability of responses to low-intensity stimulation (Brugger et al., 2011). This suggests that using knowledge of state to fine-tune the stimulation parameters could achieve reliable injection of information into the nervous system using less damaging interventions.

Knowledge of state dependence (expressed as ability to predict single-trial variations around the stimulus mean from mathematical knowledge of state dependence, Figure 2D) can also greatly improve the decoding stage of a BMI. Indeed, more information about the external variables could be obtained by simply subtracting out the estimated state-induced trial-to-trial variations of these responses. This idea is illustrated in Figure 2E, which shows the trial-averaged stimulus-evoked firing rate of a cartoon neuron to two different stimuli, as well as a single-trial response. This trial elicited a firing rate that was in-between the mean rates of these two stimuli. This intermediate-strength response could have arisen either in response to the weakest stimulus when the network was in excitable state or in response from the strongest stimulus when the network was less excitable. This ambiguity can be resolved after computing and then subtracting out the trial-to-trial variability predicted by the network state. In this example, the predicted variability was negative (indicating that the network was in a less excitable state). The subtraction of the predicted variability from the single-trial response (black upward arrows in Figure 2E) produces a “variability-discounted” response much closer to the trial-averaged response of stimulus presented in that trial (and thus much easier to decode) than the original response. Within the state dependence model of Equation (1), the state-induced variability of the responses could be discounted as follows:

(3) $$\begin{array}{l} r_{discounted}\left(s,tr\right)=\frac{r\left(s,tr\right)-b\left(\theta \right)}{g\left(\theta \right)} \end{array}$$

The subtraction of state-induced variability leads to a reduction of variability of responses at fixed stimuli (Figure 2F) of the discounted responses with respect to the original ones. Reducing variability at fixed stimulus increases stimulus discriminability (and thus information) of neural responses. Importantly, this increase of information after discounting state variability happens because taking into account the state variable reveals more tightly the relationship between stimulus and response at fixed state, and it can happen even if the state variable does not carry any information about the stimulus *per se*.

How substantial may this information gain be in real data? Safaai et al. (2015) quantified the network state as the parameters of a dynamical system (a Fitzhugh-Nagumo model, see FitzHugh, 1955) that best described the low-frequency (< 15 Hz) synchronized variations of cortical excitability before the application of a somatosensory stimulus. They subtracted from the original cortical MUA responses to the stimulus in each trial the prediction of the trial-to-trial variations of cortical firing due to state variations obtained from their dynamical system model. They found that, although the variables describing pre-stimulus state did not carry any stimulus information, the gain of sensory information obtained from the neural responses after discounting was large: it was 40% when estimating state only based on cortical ongoing activity alone, and it reached 70% when taking into account also the state of neuromodulatory nuclei releasing norepinephrine (Safaai et al., 2015). This suggests that state dependence could potentially double current information rates of decoding BMIs without increasing the size of invasive electrode arrays.

These considerations on state dependence could be incorporated into existing research directions in BMIs. Several decoding schemes, including those based on Wiener-Kolmogorov or Kalman filters non linear recurrent systems and other kinds of dynamical systems (Carmena et al., 2003; Hatsopoulos et al., 2004; Hochberg et al., 2006, 2012; Fitzsimmons et al., 2009; Sussillo et al., 2012; Kao et al., 2015) can incorporate in decoding—besides information about the “state” of the external devices to be commanded (not to be confounded with the neural state considered here) and besides other kinds of contextual modulations of neural activity such as influence of movement on sensory responses (Saleem et al., 2013; Zagha et al., 2013)—also the history of neural activity over long times scales. Thus, in principle these algorithms are well suited to be extended to include the knowledge of the state of the neural circuit. However, these studies typically included in the neural history mainly neural response components that directly carried information about the task-relevant variables to be decoded. Recent progress about state dependence of neural responses (Curto et al., 2009; Kayser et al., 2015; Safaai et al., 2015) suggests that also neural variables that represent only internal state information (such as the ongoing cortical activity or the activity of neuromodulatory nuclei) but do not directly carry information about the variables to be decoded can nevertheless greatly enhance decoding performance of BMIs, because they may allow to discount and subtract out a major source of variability. Given that activity of nuclei such as the Locus Coeruleous may be partly estimated non-invasively (Aston-Jones and Cohen, 2005), including an estimate of the activity of neuromodulatory nuclei in BMI may become useful and be feasible also in clinical applications.

It is likely that the advantage of considering state dependence may be particularly important for bidirectional BMIs when sensory and decoding operations work in a closed-loop. The ability to inject more accurate sensory feedback can lead the subject to express a more accurate motor intention, which in turn can be better decoded by discounting the state-induced variability. Similarly, brain-to-brain interfaces (Rao et al., 2014) might benefit from including state dependence as well.

## Practical challenges for exploiting state dependence for BMIs

The most promising candidate “neural state” variables that emerge from recent work typically relate to slow fluctuations of neural activity at frequencies lower than 20 Hz and that need few to several hundreds ms to be measured (Curto et al., 2009; Kayser et al., 2015; Safaai et al., 2015).

Although in some cases these low frequencies may directly carrry information about sensory or motor variables of interest—for example information about low-frequency components of dynamical natural stimuli (Rickert et al., 2005; Luo and Poeppel, 2007; Kayser et al., 2009; Belitski et al., 2010; Hall et al., 2014)—sensory or motor information in neural responses is often carried by neural firing in short time scales of few tens of ms (Panzeri et al., 2010). The potential difference of time scales for detecting task- or stimulus-informative neural variables and for detecting neural state variables poses important technological challenges for implementing state dependence in a closed-loop. In particular, electrical microstimulation produces artifacts that may mask the recorded neural signals for few ms. This issue may be addressed (O'Doherty et al., 2011) by multiplexing the recording and the electrical stimulation. The need to detect state variables operating at longer time scales calls for optimizing the time multiplexing strategy for the readout of signals at multiple time scales. The ability of the state-dependent stimulations to achieve reliable patterns even at lower current intensity (Ahmadian et al., 2011; Brugger et al., 2011) may become key to optimally integrate in real-time electrical stimulation, recoding of the neural activity and a state detector in a closed-loop bidirectional system.

If inserting knowledge of low-frequency variations in network states proves a successful strategy to improve BMIs, causal intervention of such states may become an important part of bidirectional BMIs. Thus, an interesting question is how to integrate in a close loop systems state-dependent algorithms and causal manipulation of low frequency cortical fluctuations (Thut et al., 2011; Beltramo et al., 2013).

## Author contributions

All authors listed, have made substantial, direct and intellectual contribution to the work, and approved it for publication.

## Funding

This work was supported by the European Commission (FP7-ICT-2011.9.11/284553, “SICODE,” and FP7-2007-2013/PITN-GA-2011-290011, “ABC,” and National Operational Programme for Research and Competitiveness 2007-13 PONa3_00210, “Cyber Brain”), by the MIUR Flag-Era JTC Human Brain (“Slow-Dyn”), and by the Autonomous Province of Trento (“Grandi Progetti 2012,” “ATTEND”).

### Conflict of interest statement

The authors declare that the research was conducted in the absence of any commercial or financial relationships that could be construed as a potential conflict of interest.
